# To Shrink or Not to Shrink? An Objective Assessment of Free Gracilis Muscle Volume Change in Lower-Extremity Defect Reconstruction

**DOI:** 10.3390/jcm13164811

**Published:** 2024-08-15

**Authors:** Konstantin Christoph Koban, Constanze Kuhlmann, Nikolaus Wachtel, Maximilian Hirschmann, Marc Hellweg, Konrad Wojcieck Karcz, Riccardo Enzo Giunta, Denis Ehrl

**Affiliations:** 1 Division of Hand, Plastic and Aesthetic Surgery University Hospital, LMU Munich, Marchioninistraße 15, 81377 Munich, Germany; constanze.kuhlmann@med.uni-muenchen.de (C.K.); nikolaus.wachtel@med.uni-muenchen.de (N.W.); maximilian.hirschmann@med.uni-muenchen.de (M.H.); marc.hellweg@med.uni-muenchen.de (M.H.); riccardo.giunta@med.uni-muenchen.de (R.E.G.); denis.ehrl@klinikum-nuernberg.de (D.E.); 2 Department of Plastic, Reconstructive and Hand Surgery, Burn Centre for Severe Burn Injuries, Nuremberg Clinics, University Hospital Paracelsus Medical University, 90419 Nuremberg, Germany; konrad.karcz@klinikum-nuernberg.de

**Keywords:** free muscle flap, volume change, gracilis muscle flap, lower-extremity reconstruction, three-dimensional surface imaging, objective documentation

## Abstract

**Background:** The use of free gracilis muscle flaps in reconstructive surgery of the lower leg is common practice to cover defects. However, there is still a lack of understanding of the morphometric changes that occur in the transferred muscle and area of interest over time, particularly the characteristic volume decrease that is observed over the course of the first year. This study aimed to assess volume changes in patients with free gracilis muscle flap reconstruction following infection, trauma, or malignancies of the lower extremity. **Methods:** Three-dimensional surface imaging was performed intraoperatively after 2 weeks, 6 months, and 12 months with the Vectra H2 system. A total of 31 patients were included in this study and analyzed. **Results:** There was an average volume increase of 146.67 ± 29.66% 2 weeks after reconstruction. Compared to this volume increase, there was a reduction of 108.44 ± 13.62% after 12 months (*p* < 0.05). Overall, we found a shrinkage to 85.53 ± 20.14% of the intraoperative baseline volume after 12 months. **Conclusions:** The use of non-invasive 3D surface imaging is a valuable tool for volume monitoring after free flap reconstruction of the lower extremity. The free gracilis muscle flap undergoes different phases of volume change over the first year, with the greatest influence on overall change being the development and decongestion of edema. Precise initial surgical tailoring is crucial for optimal long-term functional and cosmetic results.

## 1. Introduction

The use of free microvascular tissue transfer in reconstructive surgery has become standard practice due to its ability to provide well-perfused tissue to cover defects and a variety of free flaps, ranging from fasciocutaneous (e.g., anterolateral thigh (ALT) flap) to muscle flaps (e.g., gracilis muscle) are available for the reconstruction of the lower extremity [1,2,3]. Since its introduction in 1976, the free gracilis muscle flap is regarded as a reliable workhorse in reconstructive microsurgery and has been used to cover small- to medium-sized (<150 cm^2^) lower-extremity defects resulting from various indications, ranging from traumatic injuries to oncological defects [4,5]. Anatomically, the gracilis is spindle-shaped and long and thin in size; however, if the epimysium and perimysium are longitudinally divided, the surface of the muscle can be spread, and it can be also used to cover superficial larger defects with up to 350 cm^2^ without compromising flap vascularization [5,6,7]. Furthermore, the gracilis muscle offers a constant anatomy at the donor site, is easy to harvest, and provides the well-known advantages of excellent dead space filling and adaptation to irregular surfaces [5,8,9]. However, free muscle and especially gracilis muscle flaps are known to appear bulky at first, but undergo significant volume changes associated with a postoperative increase related to swelling and an ultimate volume decrease related to muscle atrophy that could lead to an improvement in contour over time without the need for secondary surgery [10,11,12,13,14]. Yet, volume changes vary between individuals, and long-term results are complicated by bulkiness or unpredictable atrophy of free muscle flaps and can impact the patient’s perception, as well as the postoperative treatment by means of compression treatment, shoe and orthotic fitting, and choice of clothing, affecting their quality of life [15,16]. The postoperative swelling behavior of muscle flaps is variable, but persistence of swelling over several months has been noted [14].

**Figure 1:**
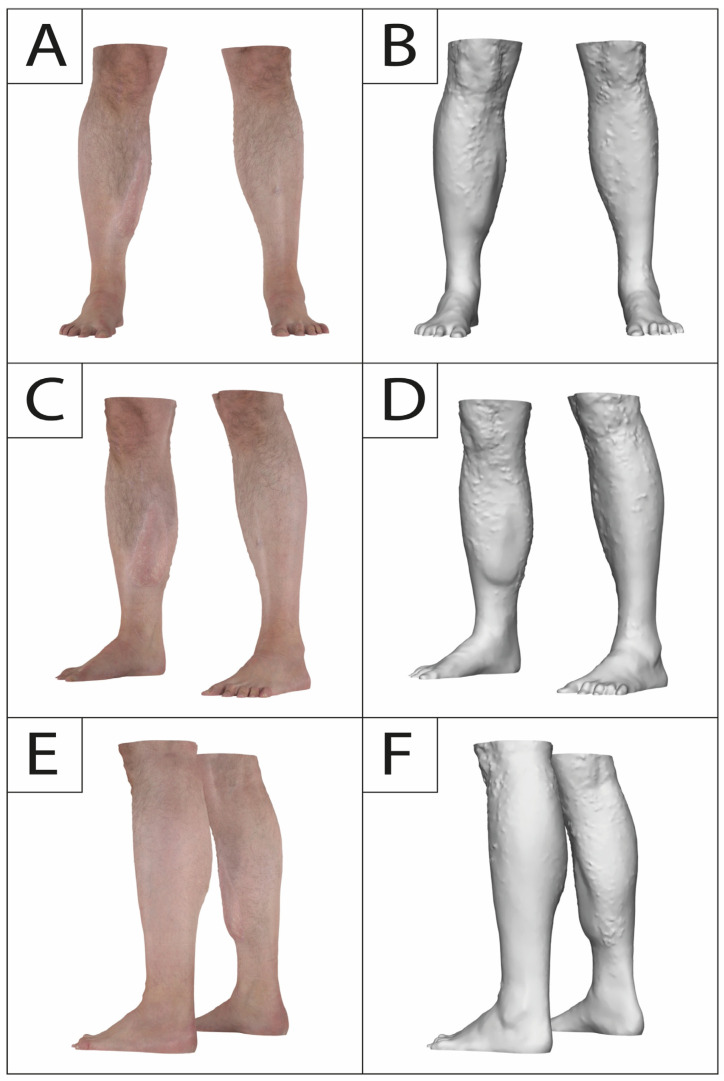
A 36-year old male presenting to our outpatient clinic complaining about muscle bulk and irregularity following a free gracilis muscle transfer to the right lower leg twelve years prior after a motorcycle accident. No secondary procedures were carried out in this interval. Frontal view (**A**,**B**), oblique frontal view (**C**,**D**), and oblique posterior view (**E**,**F**) depicting muscle bulkiness.

Previous studies (Table 1) have investigated volume atrophy after free tissue transfer using different imaging modalities, including magnet resonance imaging (MRI), computed tomography (CT), three-dimensional (3D) surface imaging, and ultrasound, as well as manual techniques, such as water displacement [12,17,18,19,20,21,22]. However, it must be noted that most studies lack an intraoperative volume as an initial baseline and are limited to small sample sizes (Table 1). In light of previous studies that reported rates for elective secondary surgery (debulking, cosmetic adjustments) of 15–17% and our own clinical experience (Figure 1), we believe that bulky gracilis muscle flaps that did not receive initial tailoring may also require follow-up procedures and do not shrink to the estimated volume as suggested before [14,23]. Thus, in this study, we aim to investigate the course of volume changes after free gracilis muscle transfer using 3D surface imaging, providing essential information for surgical planning and patient counseling, and to gain a better understanding of the amount of atrophy in denervated human muscle tissue.

## 2. Materials and Methods

### 2.1. Study Population and Surgical Technique

This study was designed as a prospective single-center study to compare the volumetric affect after free gracilis muscle flaps. The study was conducted at a level 1 hospital in Germany between May 2019 and April 2021. All free flap surgeries were performed by one microsurgeon (D.E.).

Study participation was offered to patients scheduled for reconstructive defect coverage in the lower extremity by means of a free microvascular gracilis muscle flap following infection, trauma, or malignancies. Flap elevation and the anastomoses were performed in a standardized technique. The duration of ischemia time was recorded and STSG was used to cover the muscle tissue at the recipient site. If the form or size of the defect required spreading of the gracilis muscle trough longitudinal dissection of the epi- and perimysium to achieve sufficient coverage, this was accomplished as described previously [5]. After healing, patients were advised to wear compressive garments for at least six months. A total of 35 patients were included in this study. All patients had sufficient pre- and postoperative 3D surface imaging. Flaps requiring emergent revision surgery (i.e., arterial or venous thrombosis or hematoma), partial (>10%), or complete flap loss were excluded from the study. Another exclusion criteria was patients’ loss of follow up.

Written informed consent was obtained from each participant. This study was conducted in accordance with the Declaration of Helsinki and with approval of the regional ethics committee (reference number 17-630 and 21-0475).

### 2.2. 3D Surface Imaging Device (3DSI)

The VECTRA H2 system (Canfield Scientific, Inc., Parsippany, NJ, USA) is a portable passive stereophotogrammetry 3D camera and enables up to 360° surface capturing capabilities with a single installed camera [30].

### 2.3. 3D Image Acquisition

**Figure 2:**
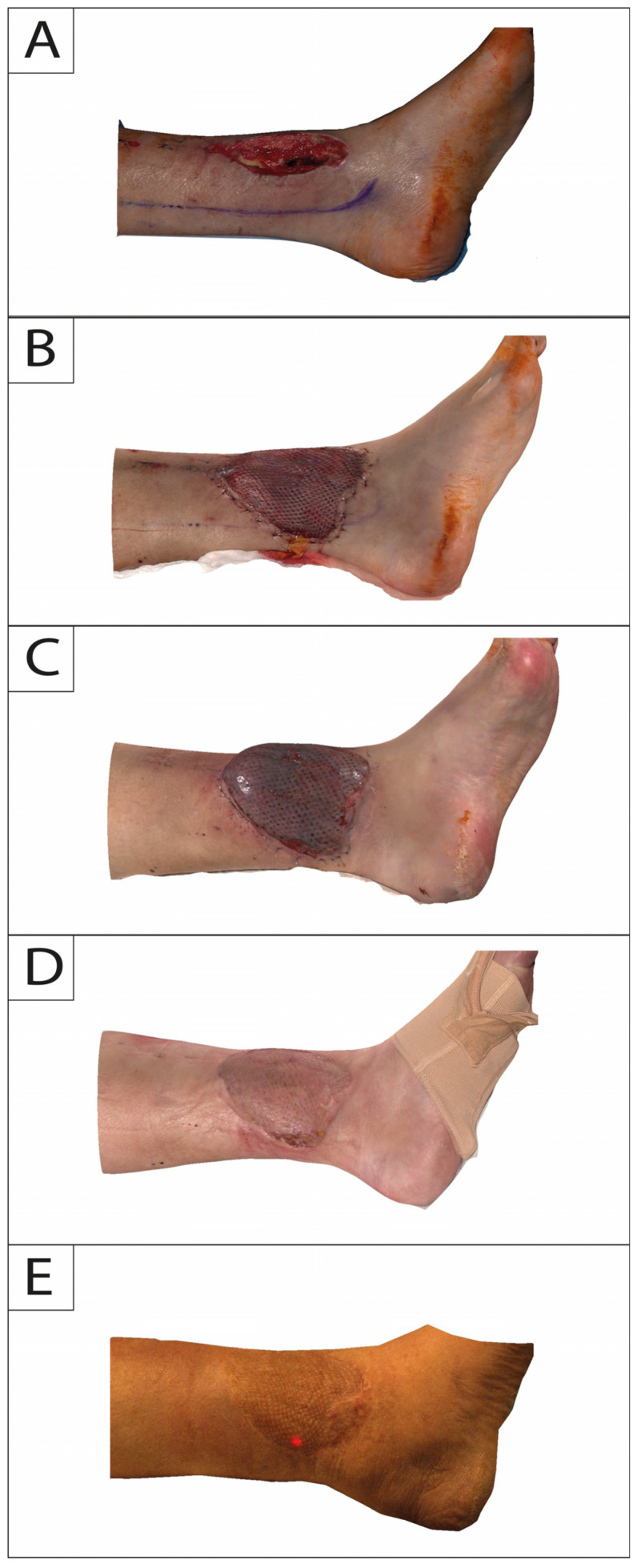
Medial profile view of a 65-year-old male suffering from exposed tibia in the lower leg following trauma. Time points of intraoperative defect (**A**): flap in-set (**B**); 2-week follow-up (**C**); 6-month follow-up (**D**); and 12-month follow-up (**E**).

**Figure 3:**
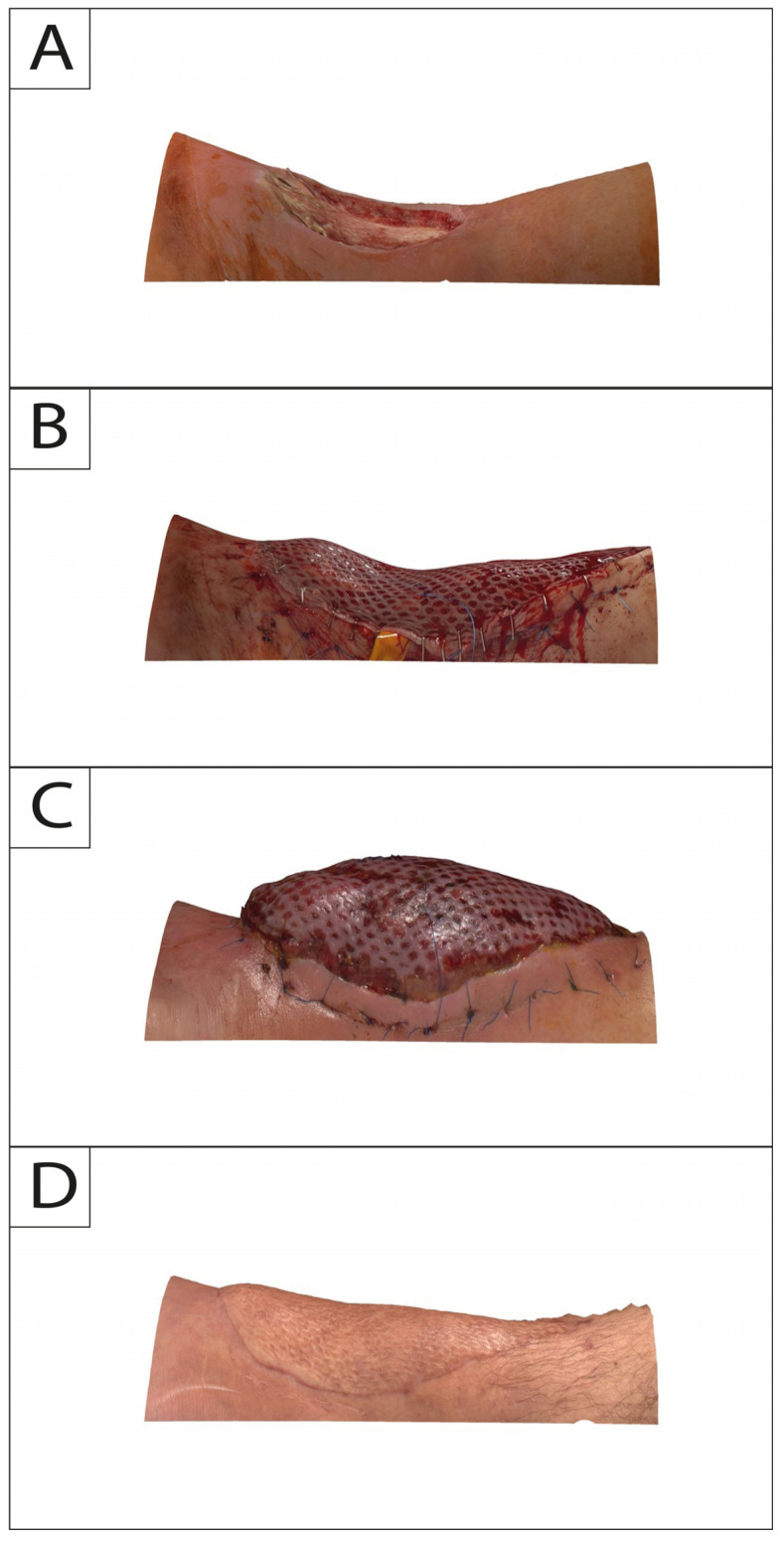
Posterior profile view of a 34-year-old male suffering from exposed tibia in the lower leg after debridement due to infection. Time points of intraoperative defect (**A**): flap in-set (**B**); 2-week follow-up (**C**); and 12-month follow-up (**D**).

In this study, 3DSI was performed for each session with Vectra H2 consecutively following a standardized protocol. The camera does not require a pre-calibrated coordinate system and features a targeting system consisting of two converging green lights projected onto the subject’s flap and lower extremity. Overlap of both lights indicates that the correct shooting distance was obtained. Regions of interest on the lower leg were captured consecutively with at least 4 separate photos directly after flap in-set (T0) and at 2-week (T1), 6-month (T2), and 12-month (T3) intervals (Figure 2 and Figure 3).

### 2.4. Data Evaluation

Based on the merging of consecutive surface acquisition, the 3D models were analyzed in the VAM application (Canfield Scientific, Inc., Parsippany, NJ, USA). Consecutive images were aligned, and between the upper and lower boundaries of the defect area volume, measurements were made to capture subsequent changes.

### 2.5. Statistical Analysis

Statistical analyses were performed using SPSS Statistics 23.00 (IBM, Armonk, NY, USA). Nonparametric testing with Friedman one-way ANOVA was used between different volume status. Difference was considered statistically significant at a probability level of ≤0.05 to guide conclusions. The figures were designed with Adobe Illustrator 2023 (Adobe Creative Cloud, Adobe Inc., San Jose, CA, USA).

## 3. Results

### 3.1. Subject Demographics

A total of 35 patients receiving free gracilis muscle flap reconstruction due to lower extremities defects were included in this prospective study (mean age: 55.2 ± 18.6 years; 17 female and 18 male). However, 4 patients were excluded from the study due to death (*n* = 1), revision surgery (arterial thrombosis *n* = 1; venous thrombosis *n* = 1), and secondary amputation of the leg (*n* = 1).

As such, 31 patients (14 female and 17 male) affected by lower-extremity defects were included in the final analysis. Study participants were between 23 and 82 years of age (mean 53.4 ± 19.6 years). The indication to perform free gracilis muscle flap reconstruction in the lower extremity was in 9 patients’ infection, in 8 patients’ trauma, and in 14 malignancies. In total, the mean time for surgery was 216 ± 35.4 min, and the mean time for ischemia was 44.9 ± 11.7 min. In 8 patients, one—and in 23 patients, two—venous anastomoses were performed. All anastomoses were hand sewn. No venous couplers were used. In 80.4% (25/31) of the study population, an arterial end-to-side anastomosis was carried out.

### 3.2. Volume Loss and Postoperative Volume Changes

**Figure 4:**
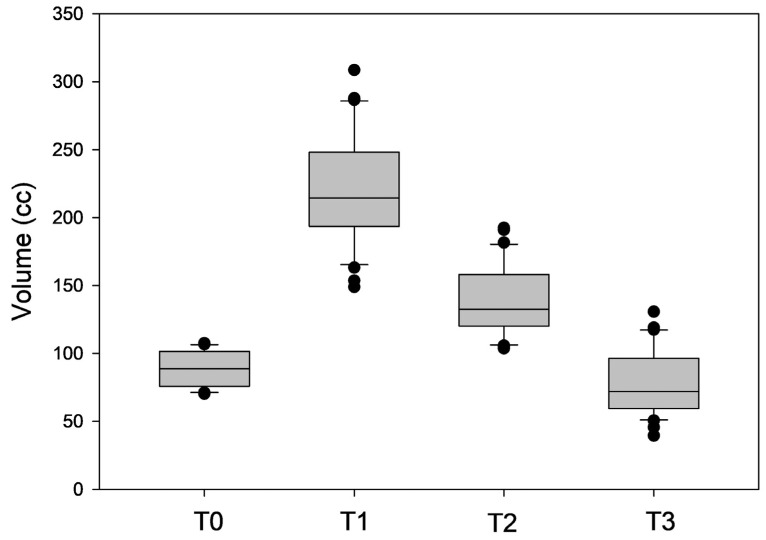
Box plot analysis depicting the measured volumes (in cc) after flap in-set (T0) and at 2-week (T1), 6-month (T2), and 12-month (T3) intervals.

Quantitative 3D surface volume change from defect to flap in-set was 89.28 ± 12.45 cc, with a volume gain of 130.86 ± 32.23 cc after two weeks. Decreases of—81.80 ± 47.22 cc and—143.12 ± 43.65 cc after 6 and 12 months, respectively, were measured (Figure 4).

There was an average volume increase of 146.67 ± 29.66% after 2 weeks (*p* < 0.05). Compared to this volume increase, there was a reduction of 58.44 ± 21.27% after 6 months (*p* < 0.05) and 108.44 ± 13.62% after 12 months (*p* < 0.05). Compared to the volume directly after flap insertion, there was a volume increase of 56.13 ± 24.86% (*p* < 0.05) after 6 months and a volume reduction of 14.47 ± 20.14% after 12 months (*p* > 0.05).

## 4. Discussion

To the best of our knowledge, we provide the largest case series measuring the volumetric changes in a specific muscle flap over a period of 12 months by providing data from 31 free gracilis muscle flaps for lower-extremity defect reconstruction with an intraoperative baseline volume that were performed by the same microsurgeon. In our study, a significant volume increase by 146.67 ± 29.66% of the initial intraoperative volume was measured after two weeks. In line with this, Salmi et al., who evaluated volumetric changes in free LDM (*n* = 7) and RAM (*n* = 3) flaps with MRI scans taken two weeks after surgery and compared them to an baseline volume that was measured via water displacement intraoperatively, reported a mean volume increase to 188% of the initial size [17]. In their study, the MRI revealed prominent edema as the cause for the massive volume increase [17]. In comparison, other studies that assessed volumetric changes in muscle or myocutaneous flaps (Table 1) lack an intraoperative status and have correlated later volumetric changes to baseline volumes (T0) that were measured between two and twelve weeks after reconstruction, neglecting the effect of postoperative swelling on muscle flap volume.

Following this phase of massive volume increase (edematous phase), we observed a mean volume reduction of 58.44 ± 21.27% after 6 month (T2) and 108.44 ± 13.62% after 12 months (T3) in comparison to T1 (2 weeks postoperatively). Accordingly, Wettstein et al. (*n* = 10) reported a volume decrease in the free gracilis muscle flap to 30 ± 4% and 19 ± 4% after 6 and 12 months, respectively, of the volume measured two weeks postoperatively with 3D laser surface scanning [19]. However, in comparison to the intraoperative status, we found a residual volume gain of 56.13 ± 24.86% after 6 months and a shrinkage of—14.47 ± 20.14% after 12 months to the initial muscle flap volume, emphasizing that the volume change that is observed over the course of the first year is mostly related to the reduction of the edema-induced swelling in the post-initial phase (decongestive phase). The above-mentioned study by Salmi et al. similarly reported a reduction to 81.4% of the baseline volume after 6 months, further supporting the notion that the volume shrinkage of muscle flaps over the first year is not as drastic as implied earlier [17,19]. Moreover, MRI scanning of the free muscle flaps revealed a reduction from moderate to slight edema over the course of six months and the beginning of fatty replacement of the muscle tissue that has not been present in earlier scans. Additionally, the thickness of the STSG improved over the course of 6 months to form a thick (1–3 mm) cover above the muscle tissue that showed perfect integration into the defect cavities [17].

Because 12 months marks the end of our observation period, our data do not provide information about the long-term development of gracilis muscle flap volume after lower-extremity reconstruction. However, previous studies observed only minor changes in volume development within myocutaneous and muscle flaps after one year [22]. In this context, Salmi et al. reported in an ultrasound- and CT-based analysis of 22 free muscle flaps, including LDM (*n* = 17), RAM (*n* = 4) and gracilis muscle (*n* = 1), that the volume and thickness of the muscle flaps approach a plateau after 9 months which was maintained during their total follow-up period (23 months) [18]. Moreover, Kang et al. measured volumetric changes of the muscle and the fat portion of pedicled myocutaneous LDM flaps for breast reconstruction (*n* = 16) annually over the course of 5 years with CT and compared the volume to the contralateral LDM. Following a significant volume decrease in the first years, from the third year, the muscle portion of the flap showed a shrinkage rate that was similar to that of normal muscle, further emphasizing that years after reconstruction only little volumetric changes can be expected in muscle flaps [11]. Similarly, Sakakibara et al. who evaluated volumetric changes in free and pedicled myocutaneous flaps for head-and-neck reconstruction (*n* = 35) over the course of 5 years observed only a little, yet significant, reduction from 54.3% after 1 year to 42% of total baseline flap volume after 5 years [26]. Due to the differences in study design and methods (e.g., type of volume assessment, baseline status) and the small sample sizes, a quantitative comparison between the volumetric developments of different types of muscle flaps based on current literature remains very limited [31]. For future research, it would be highly beneficial to conduct a study that compares the volumetric development of different muscle flaps using consistent design and methods.

On a histological level, the volumetric changes of free muscle flaps are accompanied by morphological changes that have also been observed in other denervated muscle tissues, but are not so distinct: a significant decrease in muscle fiber diameter, atrophy of type-1 fibers, and a percentual increase in type-2 fibers with fatty and fibrotic remodeling starting two weeks after reconstruction [12]. In their biopsy-based prospective histological studies, Kauhanen et al. noted a great discrepancy between individuals, and while most muscle flaps showed signs of severe atrophy, in 20 to 30%, the muscle fiber diameter returned to nearly intraoperative values, which was most likely associated with spontaneous sensory flap reinnervation [12,13,32]. In the literature, the described muscle atrophy is generally linked to the denervation of free flaps and the mechanical disuse of the muscle after flap transfer leading to tissue remodeling [13,32,33,34]. This is further underlined by the results of a prospective randomized study by Kääriäinen et al. who compared muscle atrophy in denervated to non-denervated pedicled LDM flaps for delayed unilateral breast reconstruction and revealed significantly more type-I and type-II fiber diameter atrophy when the thoracodorsal nerve was cut [34]. Yet, flap thickness was significantly reduced in both groups after 12 months, although flap thickness between the two groups did not differ significantly. The authors concluded that the loss of muscle tension alone (disuse) causes atrophy, even if innervation is intact or the flap is reinnervated. However, in combination with denervation, the disuse atrophy and fatty infiltration becomes more apparent. Additionally, muscle atrophy does not necessarily cause a volume or thickness reduction since fatty and fibrotic remodeling of the tissue occurs, and its contribution to overall flap volume development needs further investigation in the future [12,13,32,34]. Moreover, Kauhanen et al. correlated a long intraoperative ischemia time and immobilization with poorer long-term free flap muscle bulk [12,32]. Patients requiring microvascular lower-extremity reconstruction are often subject to prolonged physical inactivity due to their underlying disease (e.g., open fractures). Previous experimental and clinical studies showed that long-term bedrest is accompanied by atrophy and a shift in fiber phenotype in human muscles [33,35]. Thus, it is possible that a delay in free flap reconstruction and prolonged perioperative immobilization causes more severe atrophy in the muscle, which could also partially explain the histological discrepancies between patients in the study by Kauhanen et al. [12,32]. Another study including free and pedicled myocutaneous flaps for oral cavity reconstruction further identified postoperative infection and decreased serum albumin levels as factors that were significantly associated with reduced flap volume 6 and 12 months after insertion [24]. Nevertheless, Hiraki et al. did not distinguish between volumetric changes of the muscle and the fat portion of the myocutaneous flaps, and the above-mentioned studies are limited to a small sample sizes; thus, factors affecting volumetric changes in muscle flaps are subject to further research [24]. Moreover, the role of adjuvant radiotherapy on free flap volume and morphology remains controversial; therefore, we excluded patients who received radiation to avoid a potential bias [20,22,24,25,27,28,29,31,36,37,38,39].

Taken together with the previous histological and radiological findings from the literature, our results emphasize that the significant volume change that is observed over the course of the first year in free gracilis muscle flaps after lower-extremity reconstruction is associated with the postoperative development and the following decongestion of edema, which we identify as the main cause. Remodeling of the denervated tissue and disuse atrophy further contribute to shrinkage, yet only play a secondary role in overall volume development of the free muscle flap. It further appears, that free muscle flaps approach a stable volume between 9 and 24 months after reconstruction, and only minimal volumetric changes occur after that timepoint [11,18,26]. Those results should be considered during planning of the reconstructive procedure and for the estimation of the final surface contour. In this regard, patients should be informed about the likelihood of massive swelling of the free flap during the first weeks after surgery, followed by shrinkage to a volume that is little below the intraoperative status during the following months. Consequently, precise intraoperative flap insertion is of particular importance for the long-term cosmetic outcome of lower-extremity reconstruction with free muscle flaps. For the gracilis muscle flap, several surgical modification techniques have been described that allow a surface enlargement and thinning of the flap through microscopically aided intramuscular dissection with the removal of the perimysium [5,6,7]. A recently published study by Dornseifer et al. further suggests that negative wound pressure therapy (VAC) decreases free muscle flap volume, particularly in the early postoperative phase [40]. Although none of our patients received negative wound pressure therapy after flap insertion, its application on free muscle flaps could be considered a potential method to limit postoperative swelling and edema, and thus postoperative volume increase. This approach warrants further investigation in future studies. In this context, it would also be interesting to investigate if tailoring and inserting techniques resulting in differences in muscle tension and thickness influence the development of edema and disuse atrophy and thus short- and long-term volume development after surgery. Furthermore, the influence of the number of venous anastomoses in the operative phase and compression garments in the postoperative phase on volume development of free muscle flaps remains to be evaluated in the future.

Three-dimensional surface acquisition has been shown to be a useful addition in head-and-neck surgery [41,42], as well as breast surgery [11,43,44] for preoperative planning and postoperative outcome analysis. In particular, objective data regarding postoperative flap behavior, tissue atrophy, and follow-up procedures were obtained with several available imaging systems. The procedure also allows a comparison between different surgical techniques, such as autologous and implant-based breast reconstruction [11,44]. One of the key benefits of utilizing 3D surface measurement is its cost-effectiveness, repeatability, and non-ionizing nature. This method allows for the repeated measurement of surface changes over time without the need for radiation, in contrast to more costly imaging modalities, such as MRI [45], CT [46], and user-dependent ultrasound, as well as manual techniques, such as tape measurements [47] and water displacement. Furthermore, 3D surface imaging enables contact-free and accurate assessment of volume and surface changes in the lower extremity and muscle flaps under physiological conditions and positioning. However, it should be noted that the limitations of utilizing 3D surface scanners are primarily restricted to the detection of the surface anatomy, rendering it unable to provide insights into the underlying causes of changes in volume, such as hematoma, seroma, necrosis, edema, or chronic venous insufficiency. Especially in cases of mixed flaps, such as myocutaneous flaps, the method only allows for the calculation of overall volume change, whereas CT or MRI imaging allows for distinct measurements of each part of the flap [48].

Overall, it is important to note that absolute muscle volumes were not reported in our study design, but the preoperative defect before muscle insertion relative to the findings after flap insertion. Since we usually harvest the complete gracilis muscle belly and partially adapt it to the defect size, we see a bias with the 3D surface measurement when the baseline value is given based on the muscle volume. Furthermore, we must acknowledge the heterogeneity of our patient cohort, encompassing different age groups, underlying diseases, immobilization levels, body mass index, and gender. Recognizing these variables is crucial for contextualizing our results and applying them in clinical practice. Future research should stratify outcomes based on these factors to better understand their specific impacts on the volume development of free muscle flaps. However, our study provides a new perspective on the volume changes after free muscle transfer by assessing the volume changes immediately after the flap inset, which allows us to capture a unique snapshot of the transferred tissue before the onset of postoperative swelling and edema. Our findings provide a valuable starting point for future research in this area and have important implications for clinical practice. Further morphometric and histological studies with larger sample sizes and longer follow-up times are needed to fully understand the impact of muscle tissue remodeling on the overall functional and cosmetic outcomes of free flap reconstruction.

## 5. Conclusions

In conclusion, our results emphasize that the gracilis muscle flap undergoes different phases of volume change over the course of the first year after lower-extremity reconstruction: A characteristic volume increase within the first two weeks after insetting due to postoperative swelling, followed by a rapid volume decrease in the decongestive post-initial phase, that approaches a constant volume over the course of the following months. Development and decongestion of edema seem to have the greatest influence on overall change in muscle flap volume, while denervation- and disuse-induced tissue remodeling only secondarily contribute to further volume shrinkage. Thus, precise initial surgical tailoring of the gracilis muscle flap is required to provide an optimal functional and cosmetic long-term result and to avoid secondary surgery.

## Figures and Tables

**Table 1:** Overview of previous studies that provided data on the total volumetric development of muscle and/or myocutaneous flaps on different time points. The studies were identified by a literature search in the National Library of Medicine (https://pubmed.ncbi.nlm.nih.gov; reference date: 2 August 2024) using the keyword “muscle flap volume”; LDM = Latissimus Dorsi Muscle; RAM = Rectus Abdominis Muscle; PMMC = Pectoralis major myocutaneous flap; d = days; w = weeks; m = months.

**Ref.**	**Flap**	**Content of Flap**	**Recipient Site**	***n***	**Total Volume Assessment**	**Baseline (100%)**	**T1 (2 w) vs. Baseline**	**T2 (6 m) vs. Baseline**	**T3 (12 m) vs. Baseline**	**T4 (24 m) vs. Baseline**	**T5 (60 m) vs. Baseline**
							Mean				
[19]	Free Gracilis Muscle	Muscle	Lower extremity	10	3D Laser surface scanning	2 w post-op		30.4%	19%		
[17]	Free LDM (7), RAM (3)	Muscle	Lower extremity	10	Baseline: Water displacement; T1+2: MRI	Intraoperative	188%	81.4%			
[24]	Free LDM (10), RAM (10), PMMC (10)	Myocutaneous	Oral cavity	30	CT	1 m post-op		78.1%	71.4%		
[25]	Pedicled PMMC	Myocutaneous	Head and neck	7	CT/MRI	3 m post-op			96.9%	89.4%	
[26]	Free LDM (18); Free RAM (7); Pedicled PMMC (10)	Myocutaneous	Head and neck	35	CT	1 m post-op			54.3%		42%
[27]	Free RAM	Myocutaneous	Head and neck (Skull base)	13	CT	1 d post-op		66%	71%		
[28]	Free RAM	Myocutaneous	Reconstruction following maxillectomy	20	CT/MRI	1 m (5–30 d) post-op		67% (181–360 d post-op)			
[29]	Free RAM	Myocutaneous	Head and neck	21	MRI	1 m post-op			76.9%		
This study	Free Gracilis Muscle	Muscle	Lower extremity	31	3D surface imaging (VECTRA H2)	Intraoperative	246.7%	156.13%	85.5%		

## Data Availability

The data presented in this study are available on request from the corresponding author.
